# The association between dynamic balance and executive function: Which dynamic balance test has the strongest association with executive function? A systematic review and meta-analysis

**DOI:** 10.1007/s11910-024-01340-3

**Published:** 2024-05-11

**Authors:** Nahid Divandari, Marie‑Louise Bird, Mahdi Vakili, Shapour Jaberzadeh

**Affiliations:** 1 Monash Neuromodulation Research Unit, Department of Physiotherapy, School of Primary and Allied Health Care, Faculty of Medicine, Nursing and Health Science, Monash University, PO Box 527, Melbourne, Frankston, VIC 3199 Australia; 2 School of Health Sciences, University of Tasmania, Newnham Tasmania, 7248 Australia; 3 Mowbray Medical, Invermay, TAS 7250 Australia

**Keywords:** Executive function; attention, Working memory, Correlation Physical mobility, dynamic balance

## Abstract

**Aim:**

The aging global population poses increasing challenges related to falls and dementia. Early identification of cognitive decline, particularly before noticeable symptoms manifest, is crucial for effective intervention. This review aims to determine the dynamic balance test most closely associated with executive function, potentially serving as a biomarker for cognitive decline.

**Recent findings:**

Based on recent reviews, inhibitory control, a component of executive function, holds significance in influencing balance performance. Studies suggest that the strength of the correlation between cognition and balance tends to be domain-specific and task-specific. Despite these findings, inconclusive evidence remains regarding the connection between executive function and various dynamic balance assessments.

**Summary:**

Our review identifies a significant association between all dynamic balance tests and executive function, albeit with varying strengths. Notably, a medium effect size is observed for the Timed Up and Go and Functional Reach Test, a small effect size for balance scales, and a strong effect size for postural sway. This review underscores a clear relationship between dynamic balance task performance and executive function. Dynamic posturography holds potential as a clinical biomarker for early detection of cognitive decline, with a note of caution due to observed heterogeneity and limited studies.

**Supplementary Information:**

The online version contains supplementary material available at 10.1007/s11910-024-01340-3.

## Introduction

The global demographic landscape is predicting a substantial rise in the elderly population, expected to increase 120% from 2019 to 2050 [1]. Associated with this demographic shift, falls and dementia are significantly on the rise among older adults [2, 3]. Cognitive decline has emerged as a significant contributor to these conditions[4, 5]. Cognitive decline, often occurring before clinical diagnoses of cognitive disorders such as dementia [6, 7], provides an opportunity to detect cognitive disorders when there is minimal impairment or impact on daily function [8]. This early detection creates a window for timely intervention and tailored strategies to mitigate the progression of cognitive disorders[8]. Current approaches for identifying cognitive decline are best suited for scenarios where symptoms have already become apparent [9].

Recently, physical biomarkers have emerged as a potential identifier of pre-symptomatic cognitive decline. Reductions in physical activity happen up to nine years before clinical diagnosis of cognitive decline [10], showing the possibility of using physical fitness as a biomarker for early identification of cognitive decline before the emergence of noticeable symptoms. A crucial aspect of physical fitness is postural balance, which has emerged as a potential marker for cognitive decline, indicating a significant interplay between motor functions and cognition abilities [11]. Among various cognitive domains, executive function, a cognitive domain particularly affected by aging [12], plays a pivotal role in maintaining balance and mobility among older adults [13], particularly in dynamic balance tasks [14].

A 2020 review revealed a clear link between physical and executive function but the link between the executive function and balance association was reported as uncertain due to limited evidence [15]. A 2022 review emphasized the importance of inhibitory control, a subdomain of executive function, for balance task performance [16], but was confined to this specific aspect. A 2023 meta-analysis identified executive function, particularly in dynamic tasks, as strongly associated with balance. Remarkably, existing reviews have yet to delve into the nuanced relationship between different balance tests and executive function.

Considering this critical gap in research, our study aims to conduct a comprehensive systematic review and meta-analysis to investigate which dynamic balance test demonstrates the most robust association with executive function in older adults. To assess the authentic relationship, we concentrated on single tasks. The decline in dual-task performance in older adults may stem from either cognitive or physical changes related to aging. Additionally, given that dual-task conditions encompass cognitive elements, examining the links between balance and cognitive tasks poses difficulties due to collinearity. This intricacy adds difficulty in determining whether identified correlations result from common cognitive components or an authentic relationship between balance and cognition [17].

Given our understanding that declines in physical fitness and balance precede cognitive decline symptoms, understanding this relationship not only holds theoretical significance in elucidating the interplay between balance and cognition but also carries practical implications for developing targeted interventions and improving the quality of life for the aging population. Also, it may help in proactive healthcare early diagnosis of cognitive decline [15, 16]. Uncovering this key association furnishes an indispensable tool for healthcare professionals encountering individuals displaying signs of balance decline. It directs them to administer cognitive assessments, with a particular emphasis on executive function, thereby enabling comprehensive evaluations and tailored interventions to address both cognitive health and physical rehabilitation needs effectively.

This review aims to evaluate the evidence for the association between executive function and various dynamic balance tests in healthy older adults and to investigate which dynamic balance test has the strongest association with executive function.

## Methods

### Literature Search

#### Data Sources and Search Strategy

This review adhered to the guidelines outlined in the Preferred Reporting Items for Systematic Reviews and Meta-Analysis (PRISMA) [17]. This study is inclusive of all relevant studies exploring the correlation between dynamic balance and executive function in healthy adults aged 60 and above, until mid-December 2023. A comprehensive online search included EMBASE, MEDLINE, Scopus, PubMed, ScienceDirect, and Ovid. In addition, manual searches of reference lists from existing studies and reviews were conducted. The search terms employed encompassed postural stability OR postural sway OR balance OR mobility OR equilibrium OR physical function AND cognition OR cognitive domains OR attention OR executive function OR inhibition OR working memory OR task shift OR cognitive flexibility AND association OR correlation OR relationship. Adjustments to the keywords were made as necessary, aligning with the terminology specific to each database and mapped to Medical Subject Heading (MeSH) terms (see Appendix A). The outcomes were organized and managed using Endnote X9 (Clarivate Analytics, Philadelphia, USA) to eliminate duplicates.

#### Study selection

This study employed specific inclusion and exclusion criteria. Inclusion criteria were English-language papers published in peer-reviewed journals, cross-sectional studies examining the association between dynamic balance and executive function with data collected concurrently in a single task, and the study focused on healthy adults aged 60 and older without neurological pathological conditions. Conversely, exclusion criteria involved any cognitive impairment or pathological conditions, including dementia and its subtypes, as well as participants with neurological conditions like stroke or Parkinson’s disease., or traumatic brain injury.

Two reviewers (ND and MV) independently assessed titles and abstracts to confirm alignment with the inclusion criteria. Subsequently, full articles underwent meticulous examination by ND and Sh. J, with any discrepancies resolved through consultation with a third reviewer (MB) if required.

#### Quality Assessment and Data Extraction

The assessment of study quality involved two reviewers (authors ND and Sh. J) employing the Newcastle–Ottawa Scale, adapted for cross-sectional studies. This scale comprises eight multiple-choice questions from three broad domains: four items related to the selection of cohorts, one item related to the comparability of cohorts, and three items concerned with the outcome assessment [18]. An adapted version of the AXIS tool was used for checking the risk of bias by two reviewers (authors ND and Sh. J) [19] with disagreements resolved through consultation with a third person if needed (author MB) [19]. Data extraction, categorization, and entry into a spreadsheet were conducted, followed by verification by another reviewer (author SJ). Weekly meetings between the two reviewers (author ND and Sh. J) ensured coherence and consensus during data extraction and analysis. Disagreements were discussed and resolved via the third person if required (author MB).

For each study included in the analysis, we systematically extracted specific details, as outlined in Table 1. This includes demographic information (sample size, sex distribution, and mean age of participants) and executive function subdomains (such as working flexibility, working memory, cognitive flexibility, attention, and task-shifting). Executive function subgroup analysis within the subdomains will be undertaken if there is sufficient data to do so. Dynamic balance outcome measure tools included the Timed Up and Go Test (seconds) [20], Functional Reach Test (cm) [21], postural sway and equilibrium scores derived from postural sway assessments (score) [22], and scores from the Berg Balance Test [23], the Tinetti Balance Test [24], Fullerton Advanced Balance (FAB) scores [25], and stability index [26]. Executive function outcome tools included scores from Trail Making Test, N-back Test, Stroop Test, Verbal Fluency Test, Clock Drawing Test, Task Switching Test, Perceptual and Motor Inhibition Test, Go No Go Test, Serial Subtraction Test, Digit Span and Digit Symbol Test. The outcomes from each study, reflecting either significance or insignificance along with Pearson correlation coefficients, were extracted. To facilitate analysis, all gathered information was systematically categorized based on balance tests utilized in the respective studies, with the organized data presented in Table 1.

**Table 1:** Characteristics of the relationship between measures of executive function and dynamic balance

First Author	Number of participants	Mean Age	% Female	Balance Task	Executive Function	Association
Executive Function and dynamic balance: EF						
Kang, et al., 2022 [33]	94	72.6 ± 5.3	100%	TUG	Executive function test on Seoul Neuropsychological Screening Battery	NS r: 0.099
Jovanovic, et al., 2022 [34]	98	68.5	83.6%	TUG	Trail-Making Test	S r: 0.217
Matos, et al., 2020 [35]	28	66.7 ± 7.6	84%	TUG	N- Back Test	S r: 0.531
Netz, et al., 2018 [36]	33 M	77.2 ± 5.5	0%	TUG	MOXO DNSCPT ADHD Test, based on the Go No Go Test	S 0.653
Zettel- Watson, et al., 2017 [37]	50	69.5 ± 8.1	64%	TUG	DSB from revised Wechsler Memory Scale III	S r: 0.216
Blackwood, et al., 2015 [38]	47	74.9 ± 5.9	48.6%	TUG	Trail Making Test B	S r: 0.308
Muir-Hunter, et al., 2014 [39]	24	76.18	100%	TUG	Trail Making Test A	S r: 0.461
Kose, et al., 2016 [40]	80	75.7 ± 5.8	45%	TUG	Trail Making Test B	S r: 0.358
Kawagoe, et al., 2015 [41]	32	73.1	37.5%	TUG	N- Back Test	S r: 0.58
Berryman, et al., 2013 [42]	48	70.5 ± 5.3	58%	TUG	Stroop Test	S r: 0.565
Herman, et al., 2011 [43]	265	76.4	58%	TUG	Verbal Fluency	S r: 0.217
Hirato, et al., 2010 [44]	329	73.3	100%	TUG	∆Trail Making Test	S r: 0.34
Netz, et al., 2018 [36]	33 M	77.2 ± 5.5	0%	FRT	MOXO DNSCPT ADHD Test, based on Go No Go Test	S r: 0.530
Tsutsumimato, et al., 2013 [45]	59	88 ± 87	83%	FRT	Trail Making Test	S r: 0.10
Won, et al., 2014 [46]	164	66 ± 4.6	66.5%	FRT	Clock Drawing Test	S r: 0.201
Zettel- Watson, et al., 2017 [37]	50	69.5 ± 8.1	64%	FABS	DSB from revised Wechsler Memory Scale III	S 0.35
Muir-Hunter, et al., 2014 [39]	24	76.18	100%	FABS	Trail Making Test A	S r: 0.451
Bruce- Keller, et al., 2012 [47]	50	74.2 ± 7.8	42%	SPPB	Clock Drawing Versus	NS r: 0.02
Herman, et al., 2011 [43]	265	76.4	58	BBT	Verbal Fluency	NS r: 0.078
Rabbit, et al., 2006 [48]	69	73.2 ± 8.1	57.97%	TBT	Colour/Word Stroop Test 1	S r: 0.326
Redfern, et al., 2019 [49]	34	76 ± 4	61.7%	Postural sway	The Motor and Perceptual Inhibition Test	S r: 0.58
Redfern, et al., 2009 [50]	24	74.2 ± 4.4	50%	Postural Sway	The Motor and Perceptual Inhibition Test	S r: 0.67
Van Iresel, et al., 2008 [13]	100	80.6	50%	Postural Sway	Trail Making Test	S r: 0.893

No: Number of participants, M: male, F: Female, Number: reference of the study; TUG: Timed Up and Go Test; FRT: Functional Reach Test; TBT: Tinetti Balance Test; BBT: Berg Balance Test; FABS: Fullerton Advanced Balance Score; SPBB: Balance Score on the Short Physical Performance Battery. SLS: Single leg stance time, mCTCIB: Modified Clinical Test of Sensory Interaction on Balance. NS: Non-significant, S: Significant. Bolds are studies that had MMSE score > 24 as inclusion criteria

#### Analysis of the data

Meta-analyses were carried out using Comprehensive Meta-Analysis software, version 4, where the effect size index was computed based on Pearson’s r coefficients reported in the included studies [27]. In instances where a study presented Spearman’s rho or beta coefficient, a conversion to Pearson’s r coefficient occurred. The conversion for Spearman’s rho involved using the formula r = 2sin(rsπ/6) [28], while beta coefficients were transformed using the formula r = 0.98β + 0.05γ, with γ assigned as 1 if β ≥ 0 and 0 if β < 0 [27, 29]. For result interpretation, pooled rz values underwent retransformation to r values through an inverse Fisher z transformation: r = e(2rz—1) / e(2rz + 1), where e is approximately equal to 2.718 and rz signifies the Fisher-z-transformed r value [30]. Effect sizes were categorized based on various dynamic balance outcome measures and executive function outcome measures. Due to variations in study samples and designs, the random-effects model was employed to compute the pooled mean effect size [30, 31]. Heterogeneity across studies was tested using Q-statistics, and the I2 index was employed to assess consistency, with percentages indicating low (25%), moderate (50%), and high (75%) levels of heterogeneity [25]. Forest plots with 95% confidence intervals were generated, and standardized effect sizes were interpreted as small (0.1), medium (0.3), or large (0.5) [32]. A leave-one-out sensitivity analysis was conducted to identify studies contributing excessively to heterogeneity.

The association was considered positive if better performance on balance tests was associated with better performance on cognitive tests, even if it was reported as a negative association in the study. For instance, certain studies indicated a negative association between the time taken for the Timed Up and Go (TUG) test and the number of correct answers on cognitive tests. This implies that improved balance, reflected in a shorter TUG test duration, was linked to better cognitive outcomes, manifested by higher scores for correct answers on cognitive tests. Consequently, in such instances, the association was reversed and classified as positive within the context of this review [31].

## Results

### Studies and Participants

**Fig. 1:**
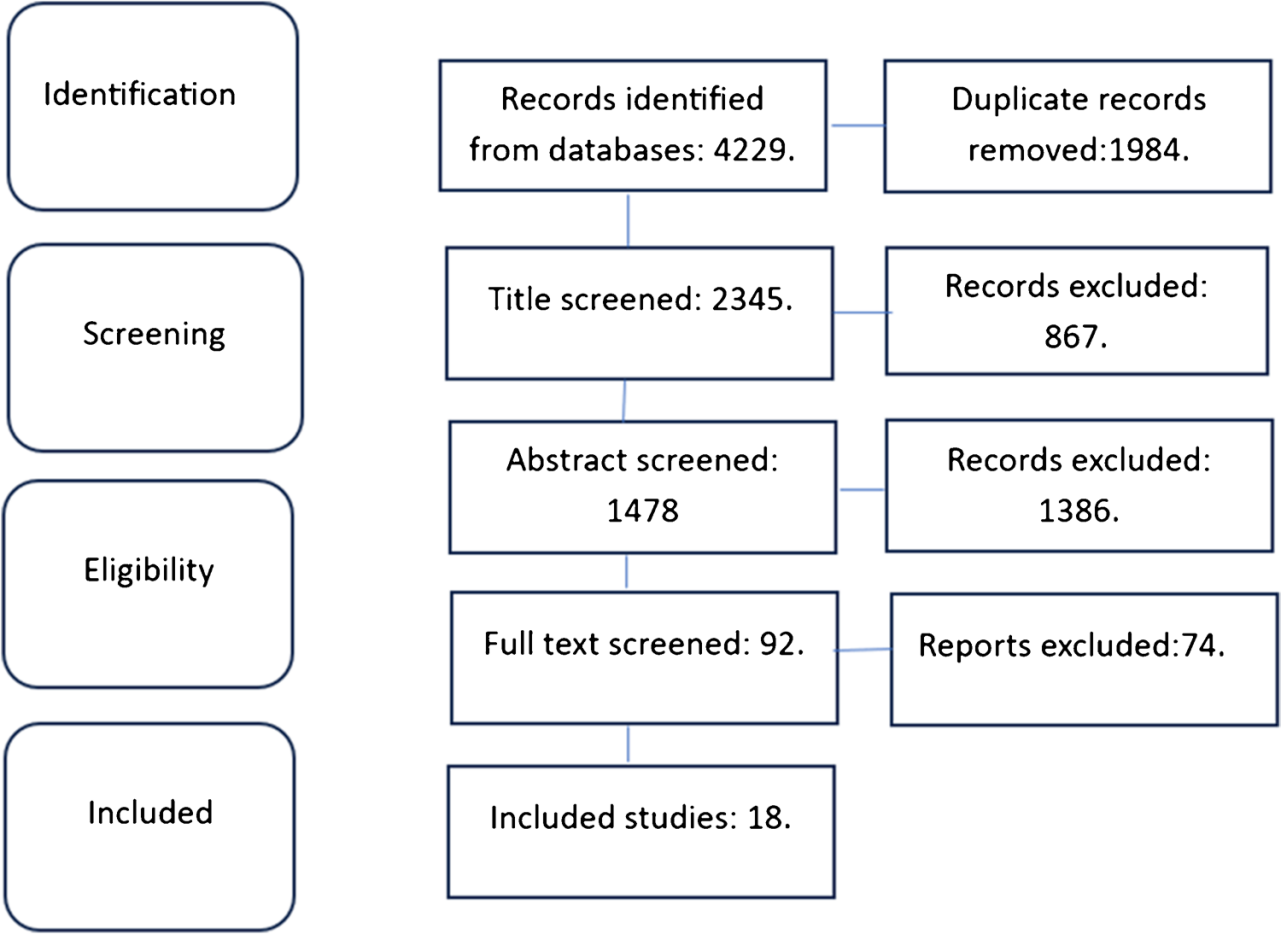
Flow diagram for study selection

Following the elimination of duplicate entries and the assessment of titles and abstracts, a total of 92 studies were initially identified. Subsequently, by applying the eligibility criteria, only 18 studies fulfilled the inclusion criteria and were ultimately incorporated into this review (Fig. 1).

The included studies examined the association of dynamic balance with executive function. Authors who did not report a correlation in cases where the association was not significant were contacted via email for clarification. The characteristics of the included studies, along with reported correlations, are summarized in Table 1.

The classification of balance tests relied on the descriptions provided in each study. In cases where the names were not specified, classifications were based on a systematic review of clinical tests of balance used in older adults and recent articles detailing subdomains of executive function and their assessments [51, 52]. If in a study there are two different types of dynamic balance tests for example TUG test and FRT, both are included in this analysis. Most frequently, the outcome measures for dynamic balance were TUG test, utilised in 10 studies.

Executive function assessments encompass subdomains such as working memory, attention, inhibition, set-shifting, verbal fluency, selective attention, visuospatial skills, and cognitive flexibility. Due to insufficient studies in each subdomain, no subgroup analysis was possible all subdomains were collectively considered under one category labelled executive function. If two tests were used within a study for executive function, the one demonstrating the strongest association with balance tests was included. The Trail-Making Test was the most frequently utilized measure of executive function, reported in eight studies, followed by the Clock-Drawing Test and Stroop tests, each employed in two studies.

### The Systematic Review Results are Summarized as Follows

#### The Association Between Executive Function Measures and Dynamic Balance Tests

Eighteen studies investigated the relationship between executive function and dynamic balance. The most used measure for dynamic balance was the TUG test time, employed in twelve studies. Postural sway, Functional Reach Test (FRT), Berg Balance Test (BBT), and Fullerton Advanced Balance Scale (FABS) were used in the remaining studies. All but two studies reported a significant association between executive function and dynamic balance, with effect sizes ranging from small to moderate (Table 1).

### Association Between Executive Function Measures and TUG

Twelve studies examined the relationship between executive function measures and TUG, outnumbering the other studies using other balance outcome measures of dynamic balance. The outcome measure of interest was for executive function among these studies Trail Making Test were used in 5 studies followed by the N-Back test for two studies. One study did not mention the type of test used for executive function (Kang, et al., 2022). All but one study (Kang, et al., 2022) reported a significant association between executive function and TUG. People with better results on the TUG test performed better in executive measurement tests.

### The Effect Size for the Correlation of Executive Function and TUG

**Fig. 2:**
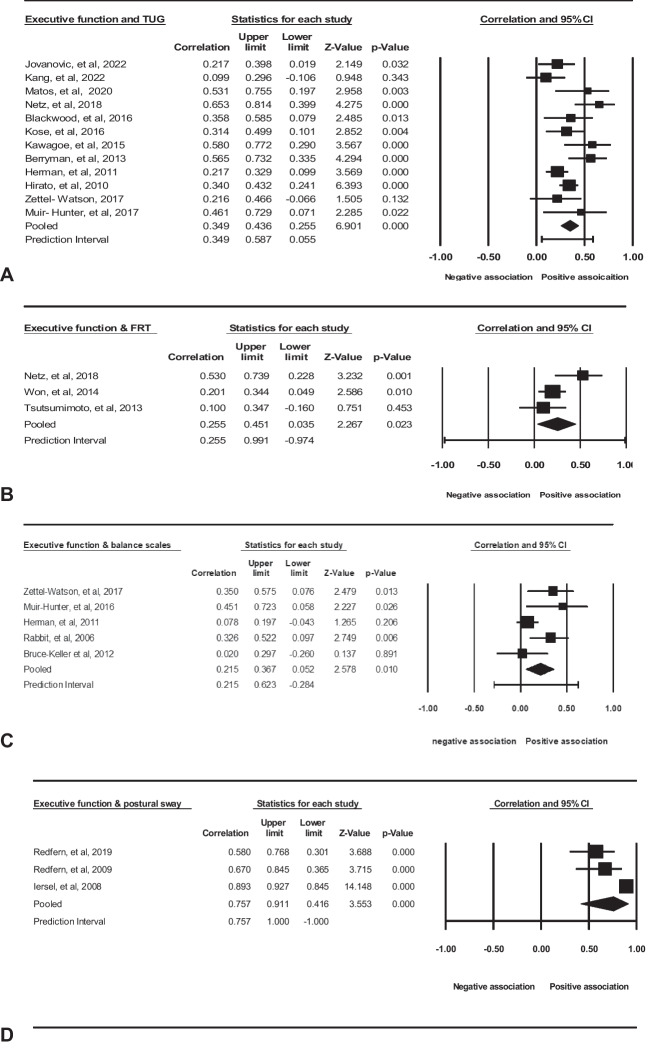
Statistical summary and forest plot of effect sizes for the association of executive function with dynamic balance tests of (A) TUG, (B) FRT, (C) postural sway, and (D) balance scales

A meta-analysis, incorporating data from 12 studies, demonstrated a moderate effect size of 0.349 (95% CI = 0.255–0.436, p = 0.000; Fig. 2, A) supporting a positive correlation between executive function and TUG performances. The findings imply that older adults with elevated executive function scores exhibited improved performance on the TUG test. However, the studies exhibited heterogeneity (Q = 25.836, p = 0.000, I^2^ = 57%). The outcome remained consistent even after the stepwise removal of individual studies (Q = 25.836, p = 0.000, I^2^ = 57%).

### Association Between Executive Function Measures and FRT

Three studies examined the relationship between executive function measures and FRT showing significant positive associations. People with better performance on executive function measurements performed better on FRT.

### The Effect Size for the Correlation of Executive Function Measures and FRT

A meta-analysis of these three studies found a moderate effect size of 0.255 (95% CI = 0.035- 0.451, *p* = 0.023; Fig. 2, B), in favour of a positive association. However, the studies were heterogeneous (Q = 4.942, *p* = 0.85, I^2^ = 50). We did not have enough studies to check the sensitivity.

### The Association Between Executive Function Measures and Balance Scales

Five studies were focused on exploring the connection between measures of executive function and different scales of balance including the Fullerton Advanced Balance Scale (FABS), and Berg Balance Scale (BBS). All studies except two revealed a significant positive association.

### The Effect Size for the Correlation of Executive Function Measures and Dynamic Balance Scales

A meta-analysis of five studies revealed a small effect size of 0.215 (95% CI = 0.052 to 0.367, *p* = 0.010; Fig. 2, C) in favour of a positive association. They were significantly heterogeneous (Q = 8.948, *p* = 0.062¸ I^2^ = 55%). The result is stable after removing the studies one by one.

### Association Between Executive Function Measures and Postural Sway

Three studies examined the relationship between executive function measures and postural sway, showing significant positive associations. People with better performance on executive function measurements performed better on dynamic balance tests measured with postural sway.

### The Effect Size for the Correlation of Executive Function Measures and Postural Sway

A meta-analysis of these three studies found a strong effect size of 0.7 (95% CI = 0.117- 0.922, *p* = 0.023; Fig. 2, D), in favour of a positive association. However, the studies were substantially heterogeneous (Q = 32.635, *p* = 0.000, I2 = 99%). We did not have enough studies to check the sensitivity.

## Discussion

The aims of this review are 1) to evaluate the evidence for associations between executive function with various dynamic balance tests in healthy older adults, and 2) to pool the individual associations between dynamic balance tests and executive function quantitatively. To the best of our knowledge, this systematic review and meta-analysis is the first to compare the relationship between executive function measures and different types of dynamic balance tests. while the primary focus of previous systematic reviews has been on the broader association between physical and cognitive function[15, 31, 53, 54], or the general association of balance and executive function [14].

### Key Findings and Consistent Positive Association:

Regarding aim 1, the findings in this review showed a consistent significant positive association between executive function with all different types of dynamic balance tests in most of the studies. The reviewed evidence shows that individuals with better dynamic balance function, no matter measured with which type of balance tests, perform better in assessments of executive function. The significant association reported in the majority of studies and the positive direction of all significant associations encouraged our conclusion. Regarding aim 2, the meta-analysis results demonstrated a significant and consistent association between executive function and all available dynamic balance tests. This uniformity across diverse tests suggests that the link between executive function and dynamic balance is not exclusive to a single test. However, it is crucial to note that the strength of this association varied among the different types of dynamic balance tests. Notably, postural sway exhibited the strongest association while dynamic balance scales showed the weakest connection. All studies examining the correlation between Timed Up and Go (TUG) and executive function, Functional Reach Test (FRT), and Posturography demonstrated a significant association between dynamic balance and executive function. However, in the case of balance scales, 2 out of 6 studies found a non-significant association between the use of balance scales and executive function. There could be different reasons for this discrepancy which include the types of participants or the ceiling effect of the tools used for the assessment of balance which is common in balance scale tests [55].

Our findings are consistent with those of previous reviews. Demnitz et al. (2016) notably identified a statistically significant small effect size in the association between balance and executive function. It is noteworthy, however, that their review was constrained to a limited scope, encompassing only three studies, and incorporating assessments of both dynamic and static balance. The nuanced nature of these results warrants careful consideration. Heaw et al. also, stated that the link between executive function and balance remains unclear due to limited evidence. As we reflect on our study, the broader context underscores the ongoing challenges in comprehensively understanding the intricacies of the relationship between cognitive processes and balance.

### Clinical Implications of Balance as a Cognitive Marker

The concept that balance might serve as an indicator of cognitive changes in old age aligns with previous studies [48]. The significant correlation noted between balance and cognition highlights the concept that balance is indicative of both physical fitness and the integrity of the nervous system [11]. Sustaining postural control necessitates the harmonious functioning of various bodily systems, and disruptions in balance may arise from alterations in these systems resulting from cognitive deterioration [56].

In our systematic review, a robust correlation between executive function and all various dynamic balance tasks was observed. Executive function, as a fundamental component of human cognition [57], plays an important role in the higher-order cognitive control of posture, and balance [58]. Deficits in executive function may lead to decompensation of higher-order gait and postural control [58]. As aging progresses, the brain undergoes substantial structural alterations, resulting in changes to the functional connectivity between networks associated with higher-order cognitive processing[59]. Extensive evidence highlights that these age-related modifications in prefrontal cortical structures are closely linked to the executive functioning observed in older adults [60]. Structural changes in prefrontal cortical structures, impact executive function and may contribute to declines in both cognitive and physical domains. Despite these age-related changes, the brain engages in compensatory mechanisms, often relying on the prefrontal cortex and higher-order cognitive processes to sustain postural control [61].

Studies have demonstrated a correlation between executive function and balance [14, 62]. Recognizing this close link between executive function, dynamic balance, and overall cognitive and physical well-being emphasizes the critical need for early identification of executive function decline using biomarkers. Safeguarding executive function becomes imperative for maintaining efficient sensorimotor processing and physical function, particularly as individuals confront the complexities of aging and potential risks of cognitive decline.

### Variation in Correlation Across Dynamic Balance Tests

The results of the meta-analysis showed that the strongest association between executive function and.

balance tests are evident in assessments conducted with posturography. Posturography provides a.

rapid and quantifiable alternative to tackle these challenges effectively [40–42]. This method involves.

the recording of the centre of pressure (COP) excursion, indicating efforts to control the centre of gravity from excessive swaying [63, 64]. It is emphasized as a more discerning indicator of instability compared to gait speed [40] and acknowledged as the gold standard for assessing balance, providing accurate and objective assessments of postural stability [41]. Clinicians need a practical and objective test for postural control that can detect cognitive decline, demonstrates validity in both experimental and clinical settings, and maintains strong reliability in repeated assessments. Posturography possesses the advantage of capturing subtle changes in balance control at rapid time scales. This characteristic solidifies its role as a robust tool for evaluating the intricate connection between executive function and balance. However, we need to be cautious interpretation of results given the observed heterogeneity, and not generalise findings across diverse populations as this study was for just healthy older adults. A review of the relationship between postural sway and cognitive domains in different populations with differing levels of cognitive capacity would add to the generalisability of our findings.

The results of the meta-analysis showed that the weakest association between executive function and balance tests belong to the balance scales tests which align with other studies. Traditional clinical balance evaluations often face challenges, such as demanding time and space, reliance on subjective scoring, and susceptibility to ceiling/floor effects [39]. Especially clinical rating scales are limited by clinicians' bias, insensitivity to mild impairments (ceiling effects), and poor reliability (65, 66). These limitations are serious concerns for clinicians and researchers who want to detect mild balance deficits or use balance as a biomarker for cognitive decline.

In examining the association between executive function measures and various dynamic balance tests, our systematic review reveals notable strengths and limitations among the included studies. A key strength lies in the diversity of dynamic balance assessments explored, encompassing TUG, FRT, BBT, and postural sway. This diversity enhances the generalizability of findings across different facets of dynamic balance. The consistent reporting of a positive association between executive function and dynamic balance in most studies adds robustness to our conclusions, supported by the inclusion of meta-analyses providing quantitative insights. However, the review is not without limitations. Heterogeneity, particularly in TUG and postural sway meta-analyses, may influence the strength of conclusions, emphasizing the need for careful consideration of contributing factors. Additionally, a limited number of studies underscores the necessity for further research to enrich the overall evidence base. While efforts were made to mitigate publication bias, its potential impact cannot be entirely dismissed. There is the need for cautious interpretation of results given the observed heterogeneity, highlighting the potential limitations in generalizing findings across diverse populations as this study was for just healthy older adults. In summary, our systematic review underscores the consistent positive association between executive function and dynamic balance, acknowledging both strengths and limitations inherent in the included studies and emphasizing avenues for future research to enhance the comprehensiveness of our understanding.

### Limitations

The findings of this review should be interpreted considering some limitations. Firstly, this study did not explore the temporal relationship between balance difficulties and cognitive deficits. Secondly, studies displayed diversity in inclusion criteria, experimental design, and sample characteristics, introducing potential confounders that may impact the generalizability of observed correlations. Thirdly, factors influencing balance, such as muscle strength and physical activity levels, were not consistently controlled across studies, potentially affecting the correlation between executive function and balance. Moreover, variations in the tests used to measure executive function introduced heterogeneity. Heterogeneity observed across studies analysing the association between executive function and dynamic balance probably was due to the diverse nature of executive function and various measurements used in studies. Additionally, the limitation of using papers published only in English could introduce bias.

### Suggestions for Future Research

To shed light on the directionality of this relationship, more longitudinal studies are needed. Further research into the mechanisms underlying the association between executive function and balance, including studies that measure brain activity during different dynamic balance tasks, is recommended. It is advisable to explore the correlation between executive function and dynamic balance in various cognitive disorders as well, as they may impact balance differently. Furthermore, there is a need for further investigation to elucidate the relationship between dynamic balance assessed through posturography and executive function. The current study was constrained by the availability of only three relevant studies. Furthermore, future research should encompass a broader spectrum by exploring these relationships within distinct subdomains of executive function and include populations with mild cognitive impairment This would contribute to a more comprehensive understanding of the nuanced associations between dynamic balance and specific facets of executive function. Lastly, an important avenue for further investigation lies in understanding the potential relationship between balance difficulties and the consequences of undiagnosed cognitive impairments. Specifically, exploring correlations with consequences such as motor vehicle accidents could have significant implications for public safety and healthcare policy. This highlights the need for future research to delve deeper into this association, informing early detection strategies and interventions to mitigate risks associated with cognitive impairments.

## Conclusion

This review sheds light on a compelling and consistently significant positive correlation between executive function and dynamic balance, which is independent of the type of balance outcome tool used., The correlation between executive function and postural sway exhibited robustness, with an effect size of 0.8, while it was moderate in TUG and FRT and weak in balance scale tests. These findings bear implications for var assessment, treatment planning, fall prevention, functional training, cognitive-motor integration, and rehabilitation outcomes. They empower clinicians to prioritize integrating the cognitive domain of executive function into interventions with dynamic balance, thereby enhancing their efficacy. Furthermore, these insights hold significance in the early identification of cognitive and balance decline, particularly in the context of aging. However, it is crucial to approach these results with caution due to the observed heterogeneity and the limited number of studies. Despite these considerations, our study provides valuable contributions to refining assessments and tailoring interventions for improved efficacy and early detection of cognitive and balance decline.

### Supplementary Information

Below is the link to the electronic supplementary material.

Supplementary file1 (DOCX 456 KB)

## Data Availability

The data supporting the findings of this study are available within the article and its supplementary materials. Additional data may be available from the corresponding author upon reasonable request.
