# Supplementary material for: The association between dynamic balance and executive function: Which dynamic balance test has the strongest association with executive function? A systematic review and meta-analysis
Source: Curr Neurol Neurosci Rep. 2024 May 11;24(6):151–61. doi: 10.1007/s11910-024-01340-3 (PMC11143012; doi:10.1007/s11910-024-01340-3)

**Appendix A:**

Search strategy and MeSH

1. Postural balance.ab,kw,ti.

2. Postural way.ab,kw,ti.

3. Equilbrium.ab,kw,ti.

4. posturography

4. 1 OR 2 OR 3

5. Cognition.ab,kw,ti.

6. Executive function.ab,kw,ti.

7. Attention.ab,kw,ti.

8. Working memory.ab,kw,ti.

9. Mental flexibility.ab,kw,ti.

10. Set shifting.ab,kw,ti.

11. Inhibition.ab,kw,ti.

12. 5 OR 6 OR 7 OR 8 OR 9 OR 10 OR 11

13. Association.ab,kw,ti.

14. Correlation.ab,kw,ti.

15. Relationship.ab,kw,ti.

16. 13 OR 14 OR 15

17. (1 OR 2 OR 3) AND (5 OR 6 OR 7 OR 8 OR 9 OR 10 OR 11) AND (13 OR 14 OR 15).

**Appendix B:**

Newcastle-Ottawa Scale adapted for cross-sectional studies.


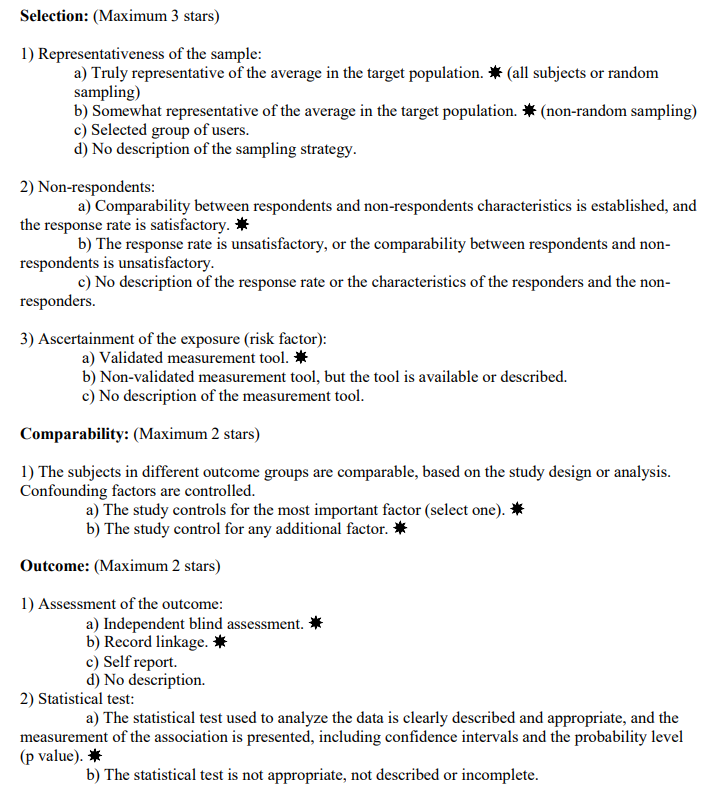


**AXIS TOOL:**


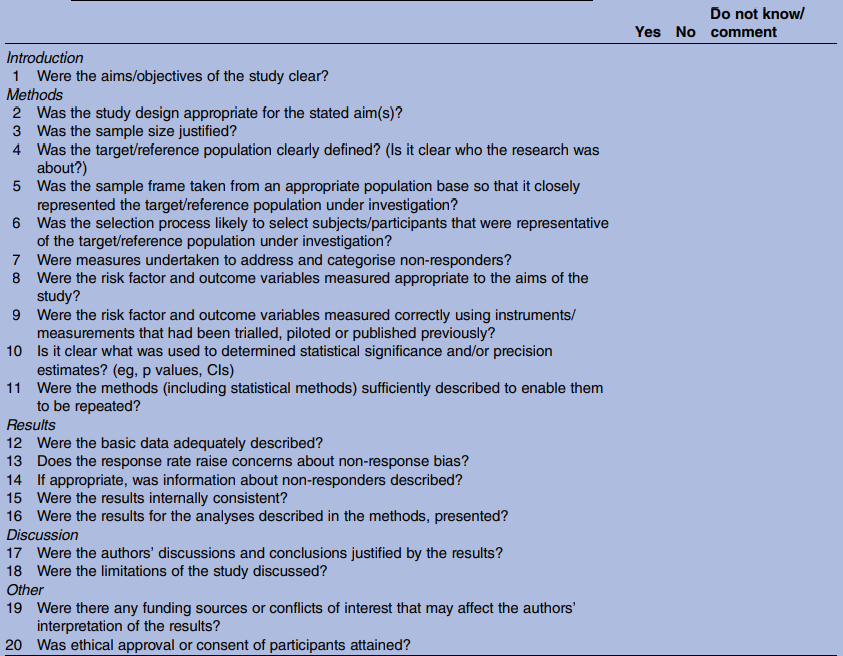

Supplement: Supplementary file 1 — Supplementary file1 (DOCX 456 KB) [file 11910_2024_1340_MOESM1_ESM.docx]
